# Case report: Recurring potassium channel complex autoimmunity-related neuropathic pain

**DOI:** 10.3389/fimmu.2024.1390171

**Published:** 2024-10-25

**Authors:** Yun-Qing Zhu, Xin-Qiao Zhou, Min Yang, Smita Horill, Zhong-Yun Wang, Jian-Jun Yang, Yin-Bing Pan, Xiao-Kai Zhou

**Affiliations:** ^1^ The First Clinical Medical College, Nanjing Medical University, Nanjing, China; ^2^ Department of Anesthesia and Perioperative Medicine, The First Affiliated Hospital with Nanjing Medical University, Nanjing, China; ^3^ Department of Anesthesiology and Surgery, The First Affiliated Hospital with Nanjing Medical University, Nanjing, China; ^4^ Department of Anesthesiology, Pain and Perioperative Medicine, The First Affiliated Hospital of Zhengzhou University, Zhengzhou, China

**Keywords:** voltage-gated potassium channel, neuropathic pain, contactin-associated protein-like 2, recurrence, antibody titer

## Abstract

Voltage-gated potassium channel (VGKC) complex autoimmunity associated with nerve hyperexcitability is an uncommon clinical spectrum. It is mostly characterized by limbic encephalitis, continuous neuromyotonia, and dysautonomia. Pain, however, has rarely been reported as the first symptom. The present study describes a case of persistent neuropathic pain as the only symptom associated with a positive serum contactin-associated protein-like 2 (CASPR2) auto-antibody in a 41-year-old female patient. Her pain was completely relieved with steroids and intravenous immunoglobulin (IVIG) therapy. Nevertheless, the pain recurred 1 year later, consistent with an immunofluorescence titer of the CASPR2 antibody. Our case shows that neuropathic pain may occur as the first and only manifestation of a VGKC complex autoimmunity disorder. VGKC antibody titers might be an indication of pain severity. Steroids coupled with IVIG are effective, but relapse may still occur.

## Introduction

Neuronal voltage-gated potassium channel (VGKC) complex hyperexcitability may be caused by an autoimmune process, causing consequences for the central nervous system, peripheral nervous system, and autonomic nervous system. This autoimmune involvement can manifest in severe forms such as limbic encephalitis, which involves inflammation of the limbic system, and Morvan syndrome, characterized by severe insomnia and neuromyotonia. It was initially described by Isaacs in 1961, with peripheral features in two patients such as persistent muscle twitching and spontaneous motor potential on needle electromyography (1). However, autoantibodies targeting neuronal contactin-associated protein-like 2 (CASPR2) were not discovered until 1999 (2). Historical reports (3, 4) indicate a broad clinical spectrum associated with CASPR2 antibodies, a component of VGKC complexes. Common clinical presentations include cognitive disturbances (26%), seizures (24%), and peripheral nerve hyperexcitability (21%) (3). The heterogeneity of these disorders contributes to the complexity of clinical symptoms. Notably, pain as an initial and sole symptom is exceedingly rare, complicating the diagnosis which typically requires the use of immunofluorescence for the antibody testing. As part of our report, we provide a description of the entire 3-year follow-up period, including diagnosis, treatment, and recurrence, so that a better understanding of this disease may be formed.

## Case report

A 41-year-old Asian female was admitted to our Pain Management Ward for the first time complaining of persistent pain in her lower limbs bilaterally for more than 6 months. It was characterized by persistent lancinating pain that worsened with cold temperatures. Her past medical history comprised of depression only. There was no record of carcinoma or any other hereditary disease in her family. Her pain had already been treated in multiple hospitals prior to this hospitalization. All hematological tests, including routine blood tests, C-reactive protein, erythrocyte sedimentation rate, liver function tests, kidney function, creatine kinase, and tumor markers, were normal. A magnetic resonance imaging (MRI) of the brain, a total spinal cord MRI, an ambulatory electroencephalogram (EEG) for 24 h, a cognitive functioning scale assessment, a pelvic MRI, and a muscle biopsy were also normal. Her only symptom of neuropathic pain, which lacked any corresponding positive test, combined with a history of depression led to a misdiagnosis. As a result, her pain was erroneously attributed to somatization disorder as a result of depression. Furthermore, the patient underwent several ineffective treatments, including antidepressant medication such as quetiapine fumarate or venlafaxine, and cognitive behavioral therapy.

**Figure 1:**
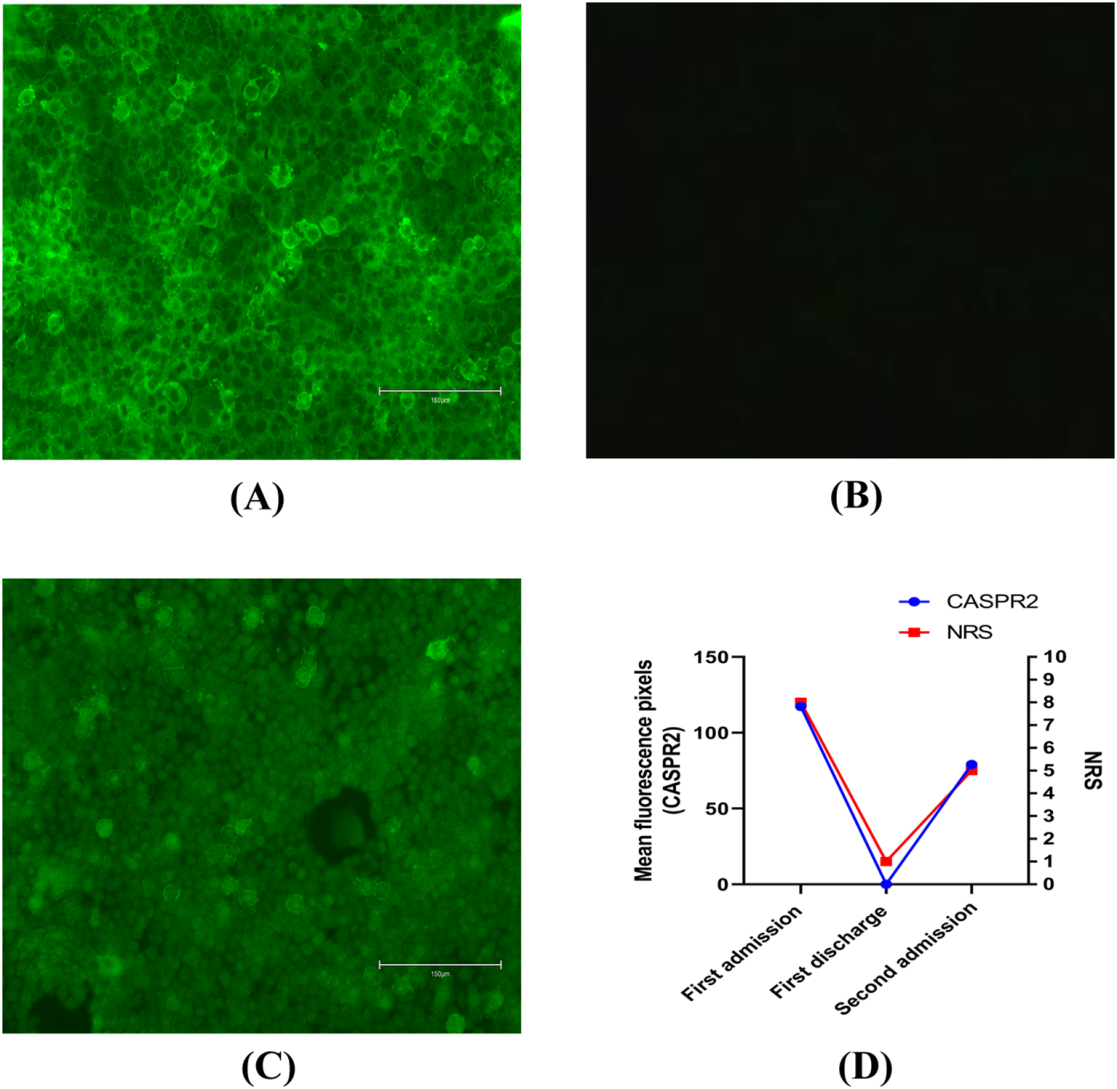
**(A)** CASPR2 antibodies were assessed by immunofluorescence in serum on first admission. Bar: 150μm. **(B)** CASPR2 antibodies were assessed by immunofluorescence in serum at first discharge. Bar: 150μm. **(C)** CASPR2 antibodies were assessed by immunofluorescence in serum on second admission. Bar: 150μm. **(D)** Changes in antibody titers of CASPR2 and numerical rating scale. All the titers were end-point titrations. Further dilutions were also performed. However, antigen-antibody reactions could also be identified by immunofluorescence after further dilutions. CASPR2, contactin-associated protein-like 2; NRS, numerical rating scale.

However, her condition deteriorated. The medication she was taking for depression had no effect on her neuropathic pain symptom. During her stay in our hospital, her muscle tone, strength, reflexes, and sensation to all modalities were all normal upon neurological examination. A needle electromyography (EMG) examination of the patient’s lower limbs including both distal and proximal muscles, such as the quadriceps femoris, gastrocnemius, and small foot muscles, was conducted by an experienced neurophysiologist, along with sensory and motor nerve conduction examinations. Her examinations and laboratory results were all within normal limits. Pregabalin and oxycodone hydrochloride extended-release tablets were prescribed initially. Her numerical rating scale (NRS) score (5) decreased from 8 points to 6 points, demonstrating a partial alleviation of her neuropathic pain. Coincidentally, during one of our regular morning rounds, we noticed brief muscle twitching in both of her legs, lasting only a few seconds. Considering her neuropathic pain and twitching movement, we performed a lumbar puncture. The cerebrospinal fluid (CSF) was clear, with a protein concentration of 530 mg/L and a chloride concentration of 118 mg/L. Her other CSF examinations, including biochemical tests, immunoglobulin G tests, and cytological tests were normal. Additionally, serum and CSF were tested for antibodies against CASPR2 and leucine-rich glioma-inactivated protein 1 (LGI1). Only CASPR2 antibodies were positive in serum (antibody titer 1:100, **Figure 1A**).

Following this, she received 5 days of intravenous immunoglobulin (IVIG) therapy (0.4mg·kg^-1^·d^-1^) (6, 7). However, there was not much improvement in her condition. Consequently, 500mg·d^-1^ methylprednisolone (7) was added sequentially to the IVIG therapy. The combination regimen resulted in better clinical relief of the intractable neuropathic pain prior to discharge, with her NRS score decreasing to 1 point. Similarly, her CASPR2 antibody levels also decreased in serum (**Figure 1B**). Prior to her discharge, a positron emission tomography-computed tomography (PET-CT) scan was performed to exclude the possibility of tumors.

She was followed up with on a regular basis. On medical advice, she gradually reduced her steroid use. Over a span of 6 months, she had completely recovered and was not on any treatment. Unfortunately, 1 year after her discharge, she complained of recurrent neuropathic pain. Approximately 20 days after the recurrence of her pain, she was referred to our hospital again with an NRS score of approximately 5 points. Based on the first result, we only tested for CASPR2 antibodies in serum. CASPR2 antibodies were again detected in serum (antibody titer 1:10, **Figure 1C**). Similar to her first hospitalization, she was treated symptomatically with pregabalin and oxycodone hydrochloride extended-release tablets. However, this did not successfully alleviate her pain. Consequently, a combination of IVIG and a high dose of methylprednisolone was administered as part of immune therapy. This relieved her pain, evidenced by an NRS score of 1 point. Moreover, a routine chest computed tomography scan was performed during her second hospitalization, which ultimately revealed lung carcinoma. Subsequently, she underwent surgical removal of the lung tumor as part of her cancer treatment. As of now, 2 years after her second discharge, the symptoms have not recurred.

## Discussion

VGKCs are widely present in the neuronal membranes of the central and peripheral nervous systems and are involved in determining the resting potential of cell membranes, controlling excitation thresholds, regulating action potential waveforms and frequency, and repolarizing depolarized membranes (6, 8). In humans, there are at least eight members of this family, which range from Kv 1.1 to Kv 1.8 (9). LGI1 and CASPR2 are two defined target antigens associated with Kv 1 (10). Between them, CASPR2 is more related to pain (30%-60%) (11) and low long-term quality of life (12). The incidence of potassium channel complex autoimmunity diseases is low, particularly when neuropathic pain is the first and only symptom. The fact that we have a complete consultation and follow-up process, including the recurrence and re-admission in our case, makes this report valuable.

The clinical spectrum of CASPR2 antibodies is broad. The complexity and variability in the symptoms caused by CASPR2 antibodies often lead to diagnostic challenges, as evidenced in this case where neuropathic pain in the lower limbs was the primary complaint and all routine tests returned negative, including needle electromyography, sensory and motor nerve conduction studies, and PET-CT scans. However, we briefly observed suspicious muscle twitching in both lower limbs on one occasion for a few seconds. As a result, we suspected that our patient had a potassium channel complex autoimmunity-related disease with pain as its first and only manifestation, as a few subtle manifestations were not evident. Our report serves as a reminder that potassium channel complex autoimmunity-related disease might be one of the reasons for unknown pain. It is often a missed diagnosis or is misdiagnosed as fibromyalgia (6%) or psychogenic causes (13%) (10).

As far as treatment is concerned, pain relief should be prioritized over immune therapy (6). Carbamazepine and phenytoin have been shown to be effective as symptomatic therapy (13). Additionally, pregabalin and gabapentin are equally beneficial (14, 15). Nevertheless, nearly 70% of patients with pain require multiple medications, of which 30% require narcotics. In our case, pregabalin and extended-release oxycodone hydrochloride tablets were combined. Considering the side effects of carbamazepine, such as leukopenia, toxic epidermal necrolysis, and Stevens–Johnson syndrome, carbamazepine was not our first choice. However, immune therapy is often necessary and effective. The available options included plasma exchange, IVIG, steroids, and oral immunosuppression. We prescribed IVIG as the first immune therapy for our patient. However, as demonstrated by previous studies (16), it was not immediately effective on its own. Consequently, we added a high dose of methylprednisolone sequentially as part of a combination therapy. Our experiences were consistent with those reported in previous studies (6). We recommend the use of IVIG as an alternative therapy for potassium channel complex autoimmunity disorders, although oral steroids can be administered concurrently or sequentially.

Regarding prognosis, the literature has reported a 25% relapse rate, some of which recurred up to 7 years after the first episode (3). Relapse was defined as a recurrence of symptoms after a complete or partial recovery, with sustained improvement for at least 2 months. According to previous studies, delayed diagnosis, inadequate initial treatment, and poor response to immunotherapy were associated with increased morbidity (17). In our case, the relapse had the same manifestations as the first episode and responded well to the same therapy, but it is also important to remember that CASRP2 antibodies may affect different parts of the nervous system compared to the initial episode. CASPR2-IgG was significantly associated with the occurrence of pain (4). Moreover, our case supports the use of CASRP2 antibody titer as a disease monitor, especially for pain severity. It also correlated with the patient’s NRS scores (**Figure 1D**). CASPR2 is a synaptic protein involved in both synapse development and maintenance. Dawes et al. (18) demonstrated Caspr2-IgG-induced mechanical pain-related hypersensitivity in mice and confirmed its pathogenicity in driving pain-related behavior, which is related to increased DRG soma excitability due to impaired Kv 1 channel function. They also showed that the CASPR2 antibody drove primary afferent excitability and pain (18), which was confirmed in our case.

Regarding comorbidity, potassium channel complex autoimmunity-related disease may be a manifestation of a paraneoplastic state by cross-reaction (4). However, the finding that patients with tumors had a similar progressive disease course as those without tumors was controversial (2). We highly recommend excluding malignancy, and PET-CT imaging should be conducted when it is highly suspected. Previous studies have reported that occult malignancies should be followed for a period of 5 years (19). In our case, no evidence of malignancy was apparent on PET-CT at first. Nevertheless, a chest CT scan during her second episode ultimately revealed lung carcinoma. Even in suspected paraneoplastic cases such as this, peripheral neuropathic pain can also be a sign of potassium channel complex autoimmunity-related disease.

Our case report provides a description of the complete 3-year follow-up period of one case of recurring potassium channel complex autoimmunity-related neuropathic pain. It is often erroneously attributed to somatization disorder as neuropathic pain might be the first and only manifestation. In addition, the antibody titers might also be an indication of pain severity. We found a correlation between CASPR2 antibody fluorescence intensity and NRS score. There are also several limitations. First, it would have been better to perform a skin biopsy during hospitalization as she had already had a muscle biopsy. Second, CSF was not tested for antibodies on her second admission. Only serum was tested due to the result on her first admission.

## Conclusion

Herein, we describe a case with recurring neuropathic pain as the first and only manifestation of a potassium channel complex autoimmunity-related disease. The heterogeneity of the disease’s clinical presentation might lead to a misdiagnosis. Furthermore, this case also emphasizes that the antibody titer of CASRP2 could be a monitor of pain severity and an indicator of a paraneoplastic state.

## Data Availability

The raw data supporting the conclusions of this article will be made available by the authors, without undue reservation.
